# CircRNA-mediated regulation of brown adipose tissue adipogenesis

**DOI:** 10.3389/fnut.2022.926024

**Published:** 2022-07-29

**Authors:** Kaiqing Liu, Xin Liu, Yaqin Deng, Zesong Li, Aifa Tang

**Affiliations:** ^1^Guangdong Provincial Key Laboratory of Systems Biology and Synthetic Biology for Urogenital Tumors, Shenzhen Key Laboratory of Genitourinary Tumor, Department of Urology, The First Affiliated Hospital of Shenzhen University, Shenzhen Second People's Hospital (Shenzhen Institute of Translational Medicine), Shenzhen, China; ^2^Shenzhen Key Laboratory of Ophthalmology, Shenzhen Eye Hospital, Affiliated Shenzhen Eye Hospital of Jinan University, Shenzhen, China

**Keywords:** browning, brown adipose tissue, white adipose tissue, *Ogdh*, circRNA

## Abstract

Adipose tissue represents a candidate target for the treatment of metabolic illnesses, such as obesity. Brown adipose tissue (BAT), an important heat source within the body, promotes metabolic health through fat consumption. Therefore, the induction of white fat browning may improve lipid metabolism. Currently, the specific roles of circRNA in BAT and white adipose tissue (WAT) remain elusive. Herein, we conducted circRNA expression profiling of mouse BAT and WAT using RNA-seq. We identified a total of 12,183 circRNAs, including 165 upregulated and 79 downregulated circRNAs between BAT and WAT. Differentially expressed (DE) circRNAs were associated with the mitochondrion, mitochondrial part, mitochondrial inner membrane, mitochondrial envelope, therefore, these circRNAs may affect the thermogenesis and lipid metabolism of BAT. Moreover, DE circRNAs were enriched in browning- and thermogenesis-related pathways, including AMPK and HIF-1 signaling. In addition, a novel circRNA, circOgdh, was found to be highly expressed in BAT, formed by back-splicing of the third and fourth exons of the *Ogdh* gene, and exhibited higher stability than linear *Ogdh*. circOgdh was mainly distributed in the cytoplasm and could sponge miR-34a-5p, upregulating the expression of *Atgl*, a key lipolysis gene, which enhanced brown adipocyte lipolysis and suppressed lipid droplet accumulation. Our findings offer in-depth knowledge of the modulatory functions of circRNAs in BAT adipogenesis.

## Introduction

An imbalance between energy intake and expenditure represents the main cause of obesity and is associated with various metabolic conditions, including non-alcoholic fatty liver disease, metabolic syndrome, cardiovascular disease, and type 2 diabetes mellitus (T2DM) (1). Reducing energy intake to treat obesity has limited effects as lipid accumulation usually persists (2). Discovery of key homeostatic factors in energy metabolism has been an important target for the prevention and treatment of obesity. In mammals, adipose tissue is divided into white adipose tissue (WAT) and brown adipose tissue (BAT) (3), and adipocytes in the former are characterized by a single lipid droplet containing triglycerides and can store excess energy, being further divided into subcutaneous and visceral adipose tissue based on location (4). As opposed to WAT, BAT is mainly distributed in the shoulders of animals, with high numbers of mitochondria and multilocular lipid droplets within adipocytes. In rodents and humans, BAT promotes energy expenditure *via* uncoupling protein 1 (UCP1)-mediated thermogenesis, thus represents a specialized heat-generating organ (5, 6). Previous studies have demonstrated that BAT activity is inversely correlated with age, percentage of body fat, and body mass index (7, 8). In addition, BAT activation and the induction of WAT browning can accelerate glycolipid uptake and reduce the requirement for insulin secretion, which may represent a new strategy to improve glucose and lipid metabolism as well as insulin resistance in obesity and T2DM (9). Conversely, BAT whitening leads to brown adipocyte death and adipose tissue inflammation (10). Taken together, brown fat plays an important role in maintaining lipid homeostasis and preventing metabolic disorders.

Circular RNAs (circRNAs) are a newly recognized class of endogenous RNAs with covalently closed loop structures that are produced through a downstream 5′ and upstream 3′ splice site connection (11, 12). CircRNAs are mainly generated through back-splicing of pre-messenger RNA and vary from <100 nt to over 4 kb in size (13). Compared with linear mRNAs, circRNAs lack 5′ caps and 3′ tails. With the development of high-throughput sequencing technology and circRNA-specific bioinformatics, a large number of mammalians circRNAs have been identified. Previous studies revealed important roles for circRNAs in various biological processes, such as adipogenesis (14), muscle development (15), and carcinogenesis (16). A recent report indicated that circRNAs have regulatory functions in obesity and adipocyte differentiation (17). However, whether there is a difference in circRNA expression between WAT and BAT as well as whether this difference has regulatory significance for the mutual transformation of WAT and BAT remains unclear.

In this study, we employed RNA-seq to comprehensively analyze the circRNA expression profiles of mouse BAT and WAT, identifying numerous differentially expressed (DE) circRNAs. Furthermore, a highly expressed circRNA circOgdh in brown adipose tissue was screened for further functional analysis. These findings not only elucidate the molecular mechanism of circOgdh during brown adipocyte differentiation, but this may provide new ideas for the treatment of metabolic diseases.

## Materials and methods

### Animals and tissue collection

Four-week-old male C57BL/6 J mice purchased from GemPharmatech (Nanjing, Jiangsu, China) were used in this study. Interscapular BAT and epididymal visceral WAT were collected as previously reported (18), immediately snap-frozen in liquid nitrogen, and stored at −80°C for RNA isolation. All animal studies were approved by the Ethics Committee of Peking University Shenzhen Hospital.

### RNA isolation, RNA-seq library preparation, and sequencing

Total RNA was isolated from BAT and WAT using TRIzol reagent (Invitrogen, Carlsbad, CA, USA) in accordance with the manufacturer's instructions. RNA purity was examined using a NanoPhotometer spectrophotometer (Implen, Westlake Village, CA, USA), and RNA integrity was assessed using the Bioanalyzer 2100 system (Agilent Technologies, Santa Clara, CA, USA). A total of 5 μg of RNA per sample were used to construct sequencing libraries. Ribosomal RNA was removed using the Ribozero™ rRNA Removal Kit (Epicenter Biotechnologies, Madison, WI, USA). Linear RNA was then digested with 3 U/μg RNase R (Epicenter Technologies) at 37°C for 30 min. RNA-seq libraries were prepared using the NEBNext Ultra Directional RNA Library Prep Kit for Illumina (NEB, Ipswich, MA, USA) as per the manufacturer's instructions. The libraries were quantified using an Agilent Bioanalyzer 2100 system and sequenced on a HiSeqXten platform with 150 bp-end reads.

### Reads alignment and identification of differentially expressed circRNAs

Raw reads were obtained from each library after RNA-seq. High-quality clean reads were then obtained by removing reads containing adapters and poly-N (defined as N is more than 5% in a read), and low-quality reads (defined as when the percentage of low-quality bases with a quality value ≤19 was more than 50%) from raw reads. At the same time, the Q20 and Q30 contents of clean reads were calculated. All subsequent analyses were based on clean reads. The clean reads obtained were aligned to the mouse reference genome using the Burrows-Wheeler Aligner-MEM with default parameters. CircRNAs were identified using CIRI2 software, which is an efficient and unbiased algorithm for *de novo* circRNA identification. The expression of identified circRNAs was normalized by reads per million mapped reads (RPM), as previously described (19). RPM was calculated using the following formula: RPM = (number of back-spliced junction reads of each circRNA)/(the total number of back-spliced junction reads) × 10^6^. DE circRNAs between BAT and WAT were identified using DEseq2 (20) with a fold change ≥ 2 and a Benjamini-Hochberg method corrected *p*-value < 0.05.

### GO and KEGG enrichment analyses of DE circRNA host genes

GO enrichment analysis of DE circRNA host genes was performed using the GOseq R package. GO terms with a corrected *p* < 0.05 were considered significantly enriched. We used KOBAS software to conduct KEGG enrichment analysis of the host genes. Pathways with a corrected *p* < 0.05 were considered significantly enriched by genes.

### Quantitative reverse-transcription polymerase chain reaction

Total RNA was isolated from BAT and WAT using TRIzol reagent (Invitrogen). First-strand cDNA synthesis was then performed using the Primescript RT Master Kit (Takara, Dalian, China) according to the manufacturer's protocol. qRT-PCR was carried out using AceQ qPCR SYBR Green Master Mix (Vazyme, Nanjing, China) on a QuantStudio 7 Flex Real-Time PCR system (Thermo Fisher Scientific, Waltham, MA, USA). The cycling parameters were set as follows: 95°C for 5 min, followed by 40 amplification cycles, each at 95°C for 10 s, and then 60°C for 30 s. All reactions were carried out in triplicate per sample. Divergent primers for circRNAs were designed according to a previous report. The primers used are listed in Supplementary Table S1. *GAPDH* was used as an internal reference gene to normalize circRNA expression levels. The relative expression levels of circRNAs were determined using the 2^−ΔΔCt^ method.

### Primary BAT cell isolation

Primary brown fat stromal vascular fraction (SVF) was obtained from 4-week-old male C57BL/6 J mice *via* the following procedure. Dissected BAT was washed by PBS, minced, and digested with collagenase I for 30 min at 37°C. Digested tissue was filtered through a 100 mm cell strainer, and the flow-through was then centrifuged at 1,000 rpm for 5 min. The pellet was washed once and resuspended in high-glucose DMEM containing 20% fetal bovine serum (FBS).

### Brown adipocyte differentiation and cell culture

To induce adipogenic differentiation of brown adipocytes, cells were plated and grown to 80% confluence. DMI hormone cocktail (1 μmol/L dexamethasone, 0.5 mmol/L 3-isobutyl-1-methylxanthine, and 5 μg/mL insulin) was added to the growth medium to induce the cell differentiation for 4 days. Cells were then kept in maintenance medium (growth medium supplemented with 5 μg/mL insulin) for an additional 8 days, with brown adipocytes differentiating into lipid droplets.

HEK-293T cells were cultured in high-glucose DMEM containing 10% FBS in a 5% CO_2_ atmosphere at 37°C.

### Luciferase reporter assay

The sequences containing predicted mi-34a-5p target sites or mutation sites of circOgdh were synthesized by TSINGKE (Nanjing, China) and cloned into the pmirGLO vector. MiR-34a-5p mimics were purchased from RiboBio (Guangzhou, China). 293T cells were seeded in 24-well plates and grown to a density of 70% confluency. Luciferase reporter vectors and either miRNA mimics or mimics-NC were transfected into cells using the RiboFECT CP Transfection Kit (RiboBio). Firefly and Renilla luciferase activity in transfected cells were quantified using the Dual-Luciferase Reporter Assay System (Promega, Madison, WI, USA) as per manufacturer instructions.

### Hematoxylin and eosin staining

WAT and BAT were isolated from mice, immediately fixed in 4% paraformaldehyde, and then paraffin-embedded, cut into 8-micron slices, and stained with Hematoxylin and eosin. Sections were deparaffinized with xylene twice for 10 min, followed by hydration in 100, 90, 80, and 70% alcohol for 5 min each. The sections were then soaked and washed in PBS solution for a total of three times, 5 min each. After 5 min of hematoxylin staining, sections were rinsed with running water. Five percent acetic acid was added for 1 min, sections were rinsed with running water, stained with eosin for 1 min, and rinsed with running water again. The sections were then subjected to dehydration in 70, 80, 90, and 100% alcohol for 10 s each, xylene for 1 min, and allowed to dry naturally in a fume hood for about 5 min before sealing. Sections were mounted onto slides with neutral gum.

### Immunohistochemistry

Immunohistochemical staining of BAT and WAT tissues was performed using SP-9002 Histostain™-Plus kits (ZSGB-BIO, Beijing, China) according to the manufacturer's protocols. Briefly, paraffin sections were dewaxed and hydrated, incubated with 3% H_2_O_2_ for 10 min at room temperature, and soaked in PBS for 10 min after washing with distilled water. Sections were then incubated with a primary antibody against UCP1 at 4°C overnight. After washing with PBS, a biotin-labeled secondary antibody working solution was added and incubated for 20 min at 37°C. Sreptavidin working solution was then added for 20 min at 37°C. Detection was carried out *via* addition of DAB chromogenic solution, and hematoxylin was used for counterstaining. Staining results was observed under the microscope.

### Oil red O staining

The cell culture medium was removed, washed twice with PBS, and fixed with ORO Fixative for 20–30 min. Cells were then washed twice with distilled water. Sixty percent isopropyl alcohol was added to soak for 5 min, followed by addition of the newly prepared ORO stain, and soaked for 10–20 min. The staining solution was then discarded, washed with water 2–5 times until there is no excess staining solution, and distilled water was added to cover the cells and observe under a microscope.

### RNase R treatment

RNase R (Genesee, Guangzhou, China) to 2.5 μg total RNA, incubated at 37°C for 30 min. The RNA expression levels of *Ogdh* and circOgdh were detected *via* qRT-PCR.

### RNA immunoprecipitation kit

The Magna RIP kit (Millipore, USA) was used to determine whether Ago2 bound circOgdh and miR-34a-5p. Briefly, 2 × 10^7^ adipocytes were lysed in RIP lysate, including 50 μL RIP lysis buffer, 0.125 μL RNase inhibitor, and 0.25 μL protein inhibitor. After centrifugation, the supernatant was collected and incubated with RIP buffer containing magnetic beads conjugated with anti-Ago2 antibody (Abcam, USA) or negative control IgG. Beads were washed with wash buffer, and the complexes were incubated with 0.1% SDS/proteinase K to remove protein. RNA was then extracted, and circOgdh as well as mir-34a-5p content enriched by magnetic beads were detected *via* RT-qPCR.

### Fluorescence *in situ* hybridization

A Cy3-labeled oligonucleotide probe for circOgdh (mmu_circ_0000231) was synthesized by RiboBio and applied for RNA FISH. The primary isolated brown adipocytes were inoculated into a Petri dish for confocal microscopy. When cell confluence reached 60%, they were fixed with 4% paraformaldehyde for 10 min and then permeated with PBS containing 0.5% Triton X-100 for 5 min. Cells were then incubated with pre-hybridization buffer for 30 min, and hybridization was carried out at 55°C for 2 h. The excess dye was washed with hybridization lotion, whereafter nuclei were stained with 1× DAPI. Cells were observed and photographed under a laser-scanning confocal microscope.

### CCK8 and EdU assays

The effect of circOgdh on cell proliferation was detected using the CCK-8 cell counting kit (Apexbio, Shanghai, China) and EdU cell proliferation kit (RiboBio, Shanghai, China). Cells (1 × 10^4^) were seeded on a 96-well plate. After 12 h, the cells were transfected with the overexpression vector or the empty vector, and then 10 μL of CCK-8 solution was added into each well of the 96-well plate at 0, 24, 48, and 72 h, respectively, and react at 37°C for 2 h. Finally, the absorbance value was measured at a wavelength 450 nm. For the EdU test, cells were cultured in 48-well plates (2 × 10^4^ cells per well). After 12 h of transfection, the cells were incubated with 100 μM EdU for 2 h. Subsequently, cells were fixed in 4% paraformaldehyde and stained with Apollo Dye Solution. DAPI was used to stain the nucleic acid within the cells. The cells were photographed under a fluorescence microscope Axio Observer (Carl Zeiss, Germany), and the number of EdU-positive cells was counted in photoshop (Adobe. us).

### Mito-tracker staining

Mito-Tracker staining was performed according to Mito-Tracker Red CMXRos (Beyotime, Shanghai. China) product instructions. Briefly, brown adipocytes were grown in confocal dishes, transfected with circOgdh overexpression vector or interfering RNA for 48 h, fixed with 4% paraformaldehyde for 10 min, washed once with PBS, and then with 100 nM Mito-Tracker Red incubate cells for 15 min. The fluorescence signals were observed under a fluorescence microscope Axio Observer (Carl Zeiss, Germany).

### Glycerol release measurement

To assess the effect of overexpression of circOgdh on lipolysis, fully differentiated mouse brown adipocytes were treated in serum-free medium for 4 h, and then cells were treated with Forskolin (Fsk) at a final concentration of 10 μM in phenol red-free DMEM complete medium for 6 h. The glycerol content in the culture medium was measured using a glycerol assay kit (Nanjing Jiancheng, Nanjing, China) and normalized to the total amount of protein in the samples.

### Mitochondrial DNA copy number

Brown adipocytes were transfected with circOgdh overexpression vector or interfering RNA for 48 h. The genomic DNA of the cells was extracted using a DNA extraction kit (Accurate biology, Hunan, China). Mitochondrial copy number was detected based on the mouse mitochondrial DNA probe method using a fluorescence quantitative PCR kit. Briefly, first, a standard curve was plotted with the log value of the positive control concentration on the horizontal axis and the Ct value on the vertical axis. Subsequently, the Ct value of the sample to be tested was used to calculate the log value of the sample RNA concentration from the standard curve, and then used to calculate its concentration. In the present study, 100 ng of each DNA sample was amplified in a final volume of 25 μL containing 1 × TaqMan^®^ Universal PCR Master Mix (Applied Biosystems). The PCR amplification was performed using a QuantStudio 7 Flex Real-Time PCR system (Thermo Fisher, Shanghai, China).

### Western blot analysis

Cells were lysed with RIPA lysis buffer mixed with 1% protease inhibitor and phosphorylase inhibitor for 20 min on ice. The lysate was collected and centrifuged at 12,000 rpm for 30 min at 4°C, and concentrations of protein were determined using a BCA Kit (Thermo Fisher, Shanghai, China). Lysate samples were separated using 4–20% SDS-PAGE, and then transferred to a BioTrace™ NT nitrocellulose membrane. Polyvinylidene difluoride (PVDV) membranes with ATGL (1:1,000, 55190-1-AP, proteintech, China), hormone-sensitive lipase (HSL) (1:1,000, 17333-1-AP, proteintech, China), Phospho-HSL (Ser660) (1:1,000, ABclonal, China) and GAPDH (1:5,000, 10494-1-AP, proteintech, China) primary antibodies were incubated overnight. After washing PVDV membrane *three* times with 1 × TBST, they were incubated with horseradish peroxidase (HRP)-conjugate secondary antibody (1:5,000, 7,074 s, CST) for 2 h. Finally, the membrane was covered with enhanced chemiluminescence reagents (Thermo Fisher, Shanghai, China) and visualized using the GeneGnome XRQ Chemical Imaging System (Gene Company Limited, Hong Kong, China). GAPDH was used as an indicator of total protein load for all western blots.

### Statistical analysis

Statistical analyses were performed using Prism 6 software (GraphPad Software, San Diego, CA, USA) and R version 3.5.2 (R Foundation for Statistical Computing, Vienna, Austria). Statistically significant differences were calculated using the one-way ANOVA or Student's *t*-test. Moreover, *p* < 0.05 was determined to be the criterion for statistically significant differences.

## Results

### Significant phenotypic differences between BAT and WAT in mice

**Figure 1:**
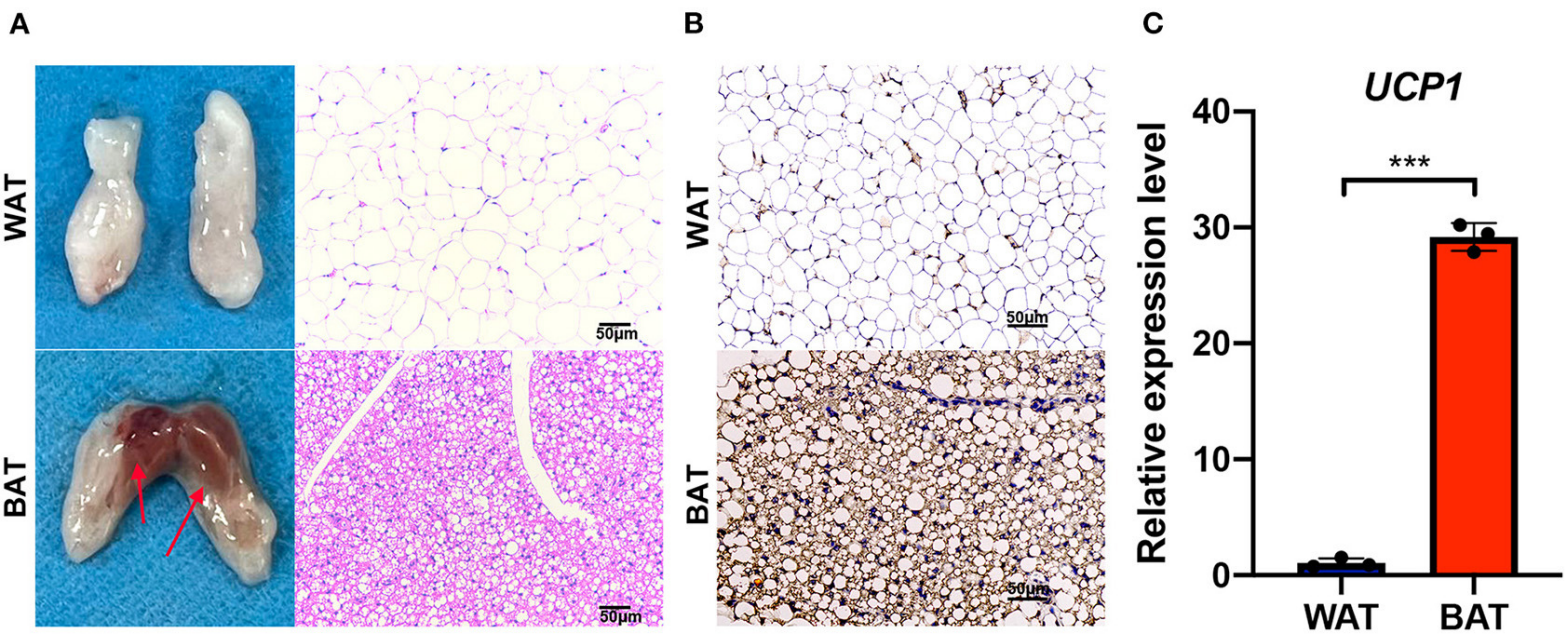
Phenotypic characterization of brown fat. **(A)** The abdominal white fat (up) and brown fat depots (down) were isolated. The right side shows the hematoxylin and eosin (HE) staining of corresponding adipose tissue, and the arrow points to the selected sample position during sequencing. **(B)** UCP1 immunohistochemical staining in white and brown fat. **(C)** Reverse transcriptase quantitative polymerase chain reaction RT-qPCR detection of *Ucp1* mRNA expression levels in white and brown fat. Scale bar, 50 μm. Values in **(C)** are the means ± SEM (*n* = 3) ***P < 0.001.

In order to identify the phenotypic differences between BAT and WAT, we isolated them from mice as previously described (21). Compared with WAT, a higher density of intracellular organelles and smaller adipocytes were found in BAT (Figure 1A). The expression of the brown fat marker UCP1 was significantly higher in BAT than in WAT as assessed by immunohistochemical (IHC) staining and reverse transcriptase quantitative polymerase chain reaction (RT-qPCR) (Figures 1B,C).

### CircRNA expression profiling in BAT and WAT

**Figure 2:**
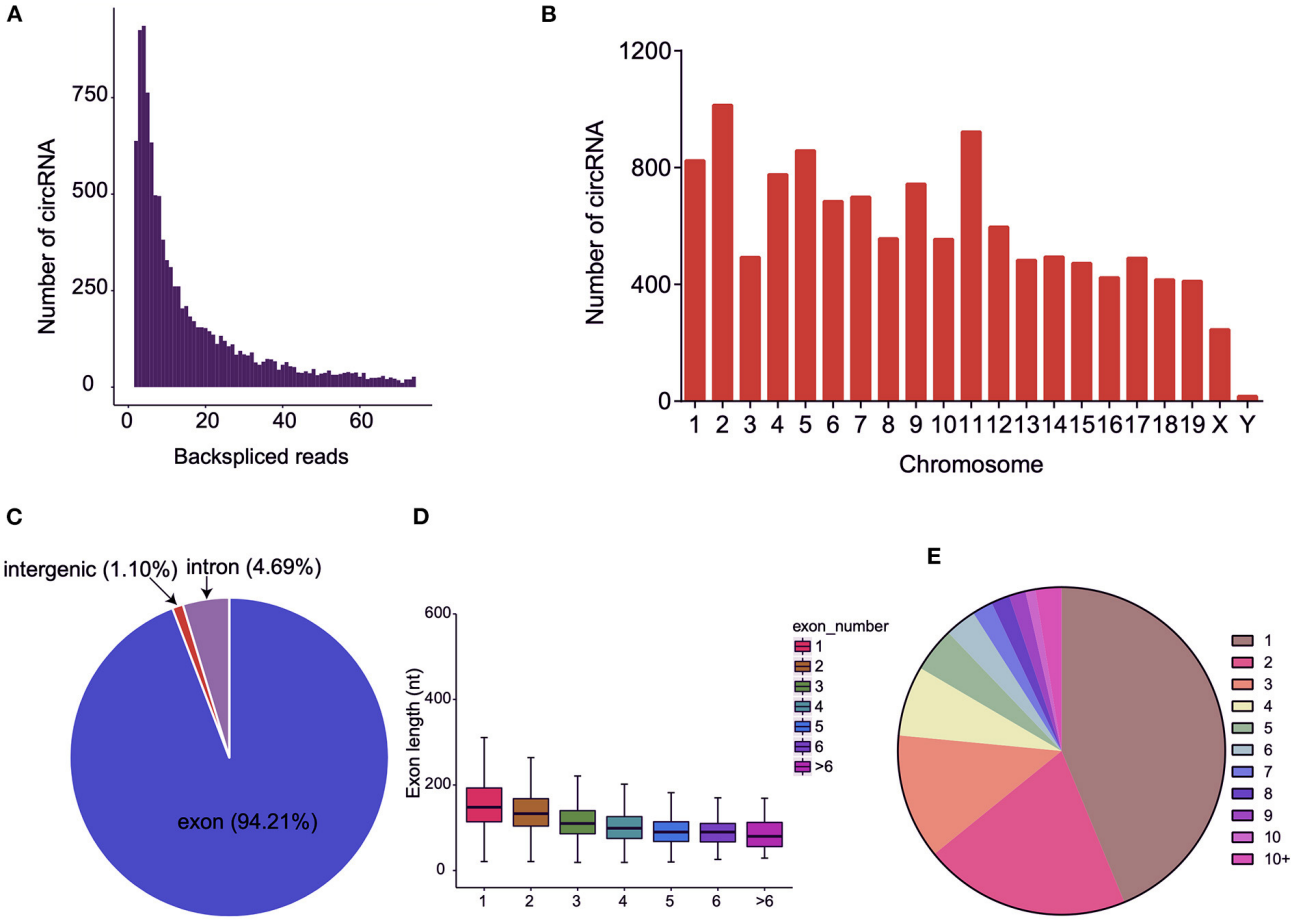
Characteristics of circular RNAs (circRNAs) in brown adipose tissue (BAT) and white adipose tissue (WAT). **(A)** The number of circRNAs and back-spliced reads identified in three BAT and WAT samples. **(B)** Distribution of the identified circRNAs on different chromosomes. **(C)** Genomic origin of the identified circRNAs. **(D)** Distributions of exon length for the exon-derived circRNAs. **(E)** Number of circRNAs generated from a single gene.

We utilized RNA-seq to ascertain the circRNA expression profiles of three pairs of BAT and WAT. In total, 343,924,644 raw reads were obtained from the six sequencing libraries (Supplementary Table S2), and 322,773,748 clean reads were finally obtained after filtering adapter contaminations containing poly-N and low-quality reads. Subsequently, 299,089,928 reads were mapped to the mouse reference genome. Detailed information on each sample is listed in Supplementary Tables S2, S3. In this study, 12,183 circRNAs that contained at least two junction reads were identified from BAT and WAT (Supplementary Table S4; Figure 2A). Among these circRNAs, 4,706 were matched in the circBase database, and 7,477 circRNAs were identified as novel (Supplementary Table S4). Distribution analysis of the identified circRNAs indicated that the number differed between chromosomes (Figure 2B). We further annotated circRNAs and found that most were derived from protein-coding exons (Figure 2C). The exon length of single-exon circRNAs was longer than that of multiple-exon circRNAs (Figure 2D). The host genes of circRNAs mostly gave rise to a single circRNA (Figure 2E).

### Identification of DE circRNAs between BAT and WAT

**Figure 3:**
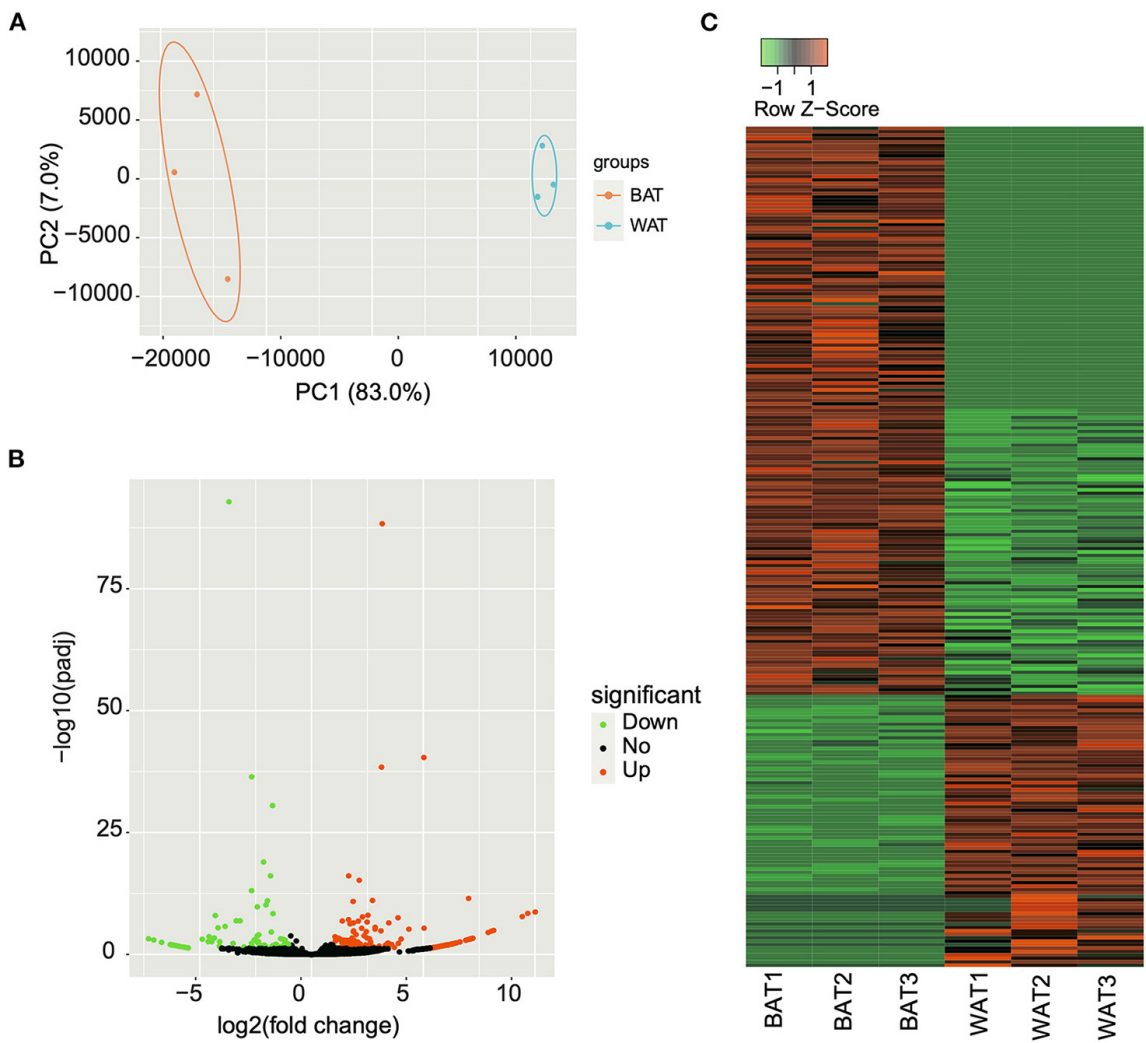
Differentially expressed (DE) circular RNAs (circRNAs) between brown adipose tissue (BAT) and white adipose tissue (WAT). **(A)** Principal component analysis of the relative distances between BAT and WAT samples. **(B)** Volcano plot of DE circRNAs in BAT compared with WAT. The green, black, and red points represent downregulated, unchanged, and upregulated circRNAs, respectively. **(C)** Heatmap showing the expression changes of DE circRNAs in BAT and WAT.

The expression levels of all identified circRNAs were calculated as RPM value. Principal component analysis was used to determine the similarity in circRNA expression profiles between BAT and WAT. The results indicated that the three individuals from the same group had a highly similar gene expression pattern (Figure 3A). After strict differential expression analysis, a total of 244 DE circRNAs were identified between BAT and WAT, including 165 upregulated and 79 downregulated (Supplementary Table S5; Figures 3B,C).

### Validation of the identified DE circRNAs

**Figure 4:**
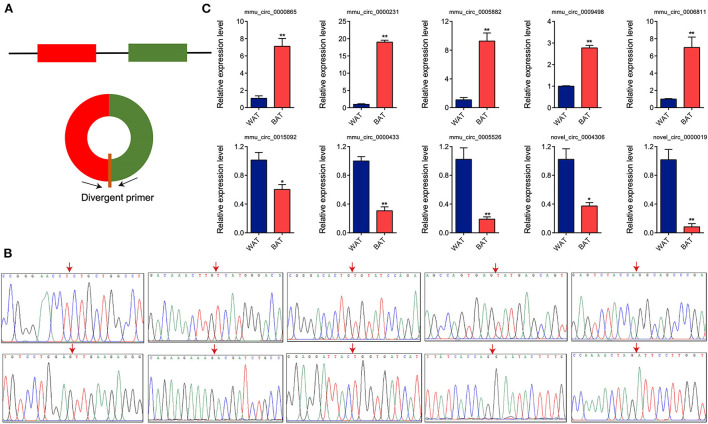
Validation of differentially expressed circular RNAs (circRNAs) through Sanger sequencing and RT-qPCR. **(A)** Schematic illustration of the divergent primer design. **(B)** Sanger sequencing of the polymerase chain reaction products. Arrow represents back-spliced junction. **(C)** Relative expression levels of 10 randomly selected circRNAs. Values in **(C)** are means ± SEM (*n* = 3). *P < 0.05, **P < 0.01.

To validate RNA-seq differential expression data, we randomly selected 10 DE circRNAs (five upregulated and five downregulated) and detected their expression levels *via* RT-qPCR. We designed divergent primers based on circRNA sequences (Figure 4A). PCR products were obtained using these divergent primers, and the junction sequences were confirmed through Sanger sequencing. The 10 circRNAs generated *via* head-to-tail splicing and junction sequences were consistent with those obtained from RNA-seq (Figure 4B). We further utilized RT-qPCR to determine the expression levels of these circRNAs, observing results consistent with RNA-seq data (Figure 4C), indicating that the results of RNA-seq are reliable.

### GO and KEGG functional enrichment analysis of DE circRNAs

**Figure 5:**
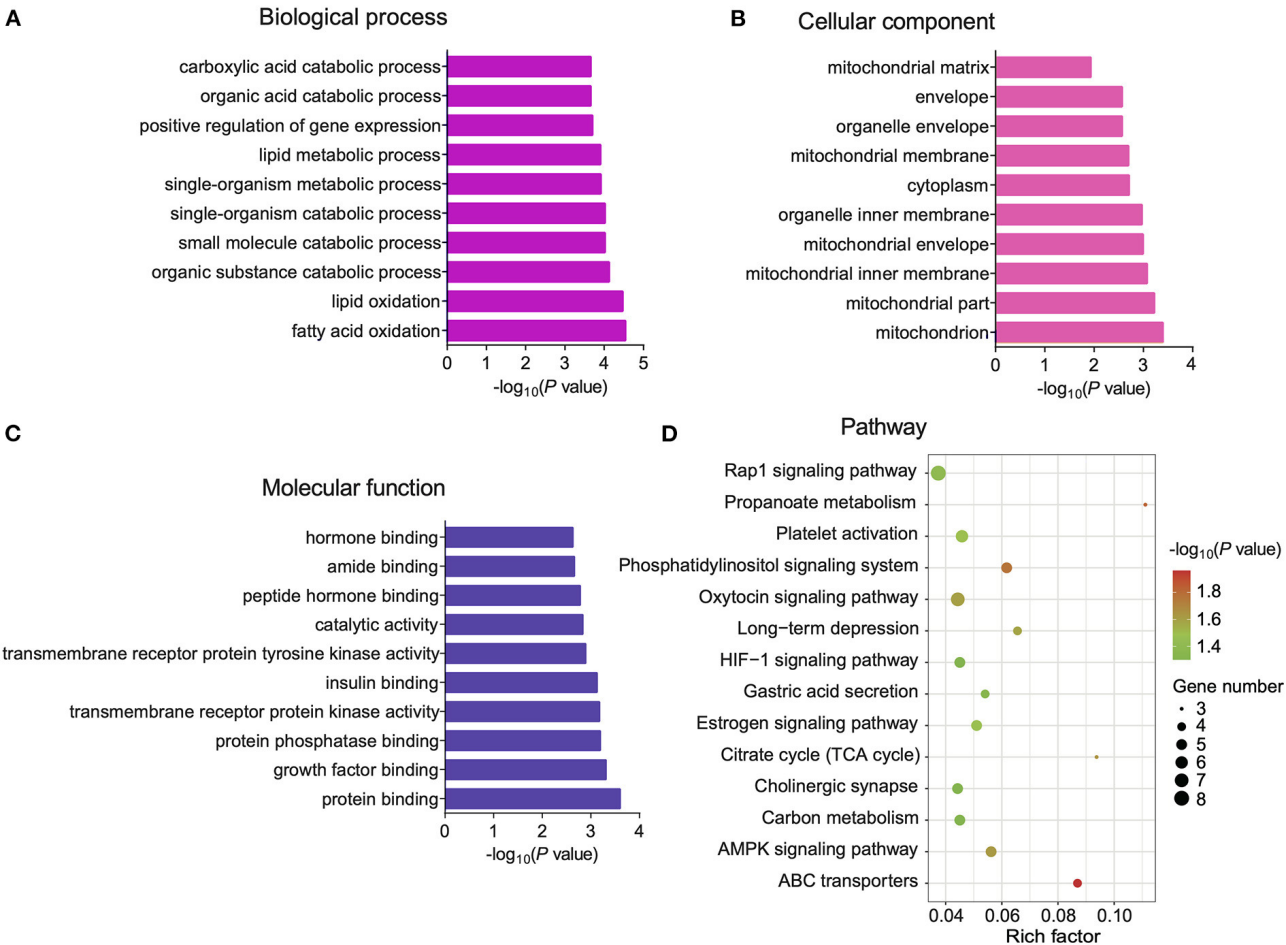
Gene Ontology (GO) and Kyoto Encyclopedia of Genes and Genomes (KEGG) enrichment analyses of differentially expressed (DE) circular RNA (circRNA) host genes. **(A–C)** The top 10 GO terms in the biological process (BP), cellular component (CC), and molecular function (MF) categories, respectively. **(D)** Significantly enriched pathways for DE cricRNA host genes.

Previous studies have reported that circRNAs affect the expression level of host gene transcripts by competing with pre-mRNA splicing (22). To explore the regulatory mechanisms of DE circRNAs in BAT and WAT, we subjected circRNA host genes to GO and KEGG enrichment analyses. The 10 most significantly enriched GO terms in biological process, cellular component, and molecular function categories are listed in Figures 5A–C. Interestingly, significantly enriched GO terms, including mitochondria, the mitochondrial inner membrane, and the mitochondrial envelope, were associated with BAT thermogenesis (Figure 5B). Furthermore, KEGG enrichment analysis results indicated that a total of 14 pathways were significantly enriched, including ABC transporters as well as the hypoxia-inducible factor-1 (HIF-1) and AMP-activated protein kinase (AMPK) signaling pathways (Figure 5D).

### CircOgdh regulates the proliferation and differentiation of brown fat cells

**Figure 6:**
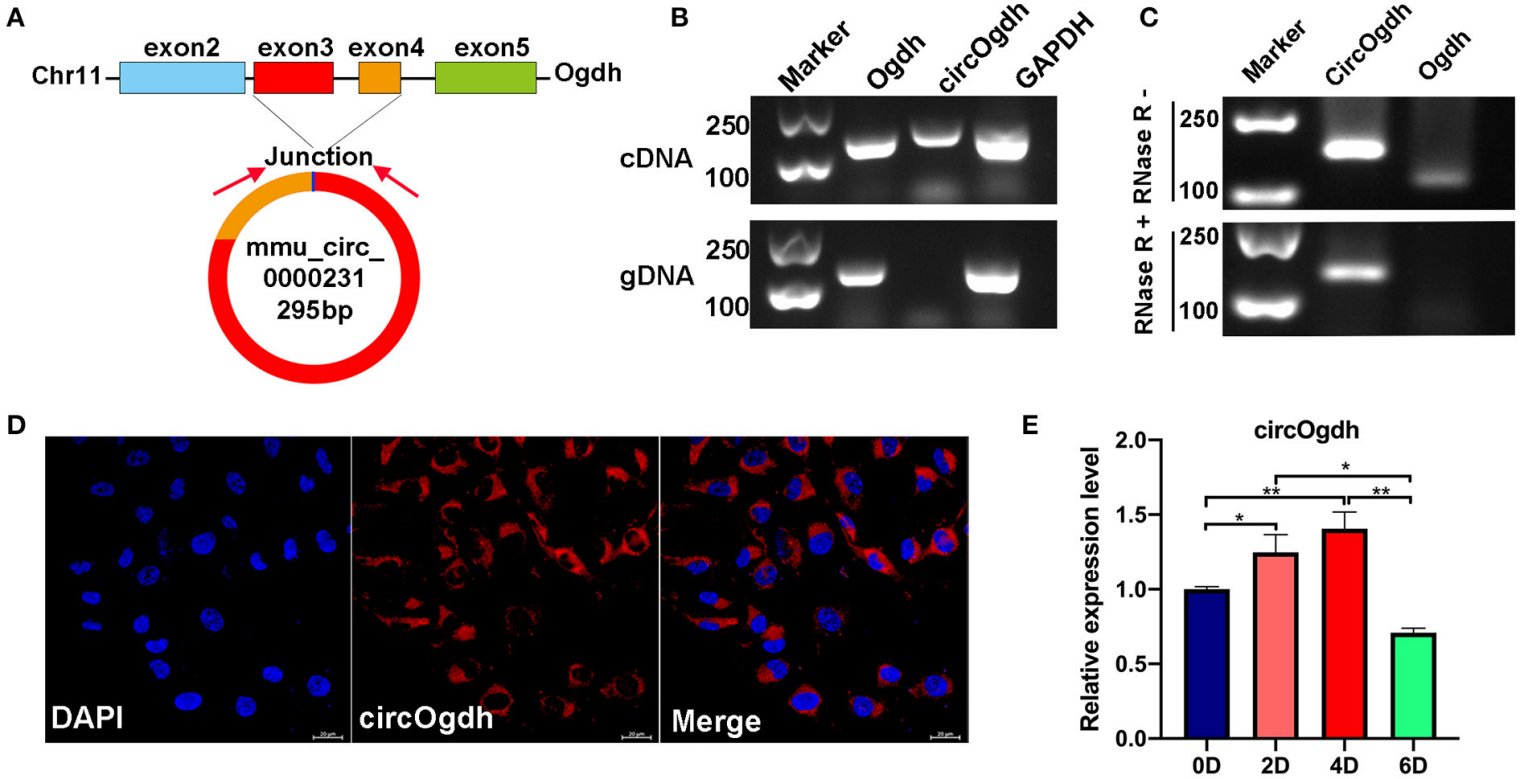
Expression and biological functions of circOgdh. **(A)** Genomic locus of circOgdh gene. circOgdh is produced at the *Ogdh* gene (NM_001252282.1) locus containing exons 3-4. **(B)** PCR analysis for circOgdh and its linear isoform *Ogdh* in cDNA and genomic DNA (gDNA). **(C)** The RNase tolerance of circOgdh was verified through RNase R treatment and RT-qPCR. **(D)** Cellular localization of circOgdh was detected *via* FISH. Nuclei were labeled with DAPI dye. The majority of circOgdh was labeled with Cy3 (Red). Scale bar, 20 μm. **(E)** RT-qPCR was used to detect the expression of CircOgdh during mouse brown adipocytes differentiation. The values are means ± SEM (*n* = 3), *P < 0.05, **P < 0.01.

We found that mmu_circ_0000231 is highly expressed in BAT, but research on this circRNA is limited. We determined that mmu_circ_0000231 is generated from the 3rd and 4th exons of the *Odgh* gene, measuring 295 bp in length. We named it circOgdh (Figure 6A) and then conducted Sanger sequencing to confirm the back-splicing junctions of circOgdh (Figure 4B). PCR analysis indicated that divergent primers could amplify circOgdh from cDNA, but not from gDNA (Figure 6B). RNase R tolerance experiments indicated that circOgdh is more resistant to RNase R than linear RNA (Figure 6C). In addition, we found that circOgdh was mainly localized in the cytoplasm of brown adipocytes based on FISH experiments (Figure 6D). The expression pattern of circOgdh during brown adipocyte differentiation showed that it was up-regulated in the early stages of differentiation and decreased on the 6th day of differentiation (Figure 6E).

### CircOgdh regulates the proliferation and differentiation of brown fat cells

**Figure 7:**
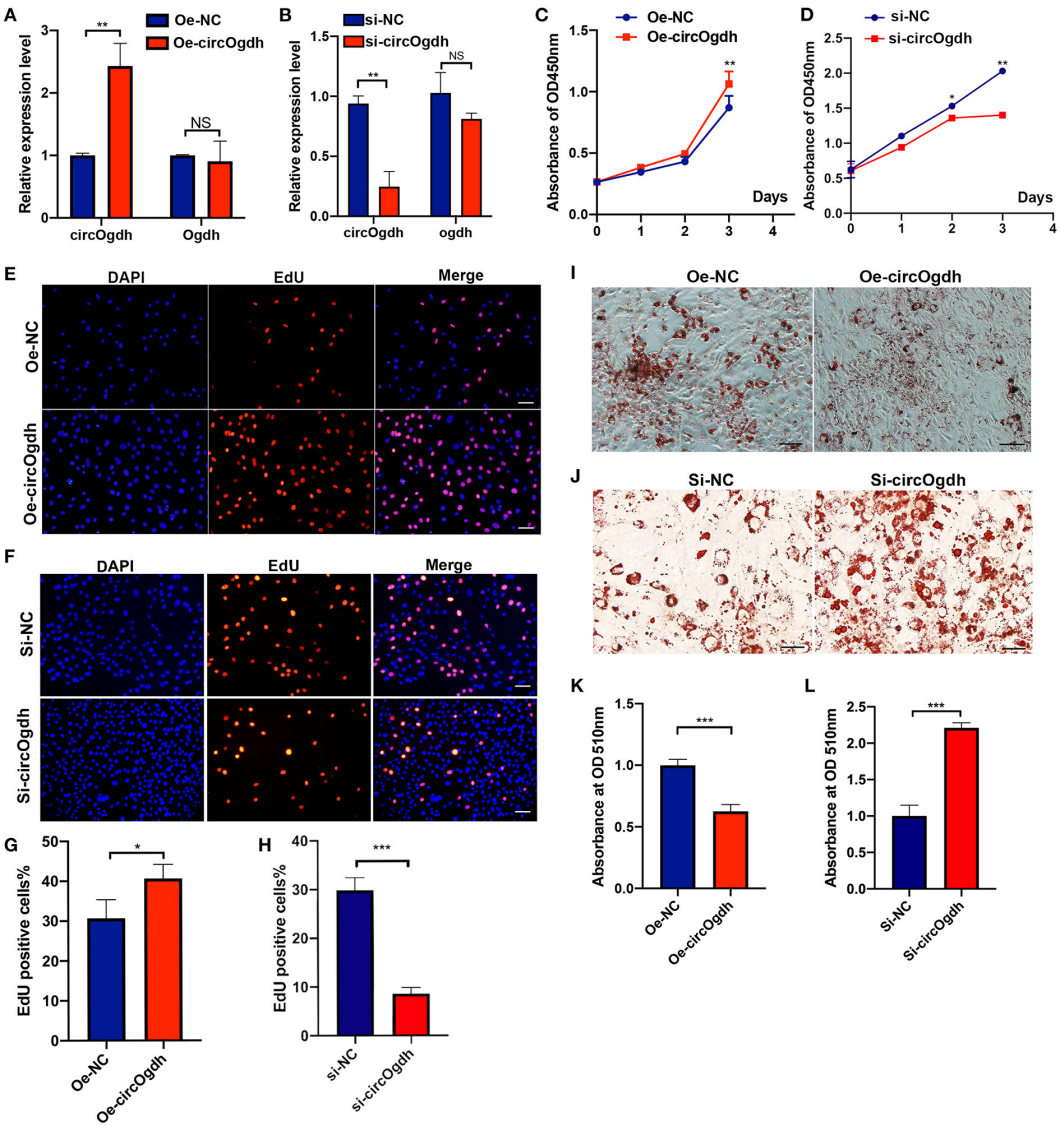
CircOgdh promotes brown fat proliferation and reduces lipid droplet accumulation. **(A,B)** After transfection with Oe-NC, Oe-circOgdh, si-NC, and si-circOgdh in brown adipocytes, RT-qPCR was adopted to detect the levels of circOgdh and *Ogdh* expression. **(C,E,G)** CCK8 and EdU staining were also performed to evaluate the proliferative capacity of BAT cells under circOgdh overexpression, **(D,F,H)**, and to determine the effect of circOgdh knockdown on cell proliferation. Scale bar, 20 μm. **(I,J)** Oil Red O staining was performed to assess adipogenic differentiation of brown adipocytes. Scale bar, 20 μm. **(K,L)** The extracted Oil Red O dye was quantified based on the absorbance values at 510 nm. Values in **(C,D,G,H,K,L)** are means ± SEM (*n* = 3), *P < 0.05, **P < 0.01, ***P < 0.001, NS, Not Statistically Significant.

To investigate the role of circOgdh in BAT development, we overexpressed it in brown adipocytes (Figure 7A). CircOgdh could promote adipocyte proliferation significantly (Figures 7C,E,G). The overexpression of circOgdh also significantly reduced lipid contents in brown adipocytes (Figures 7I,K). Conversely, when we knocked down the circOgdh expression (Figure 7B), the proliferation of brown adipocytes was inhibited (Figures 7D,F,H), and when the cells were induced to perform adipogenic differentiation, lipid droplet contents in the cells were more abundant (Figures 7J,L). The results suggest that circOgdh could promote brown fat proliferation while reducing lipid deposition.

### CircOgdh reduces lipid deposition by promoting lipolysis

**Figure 8:**
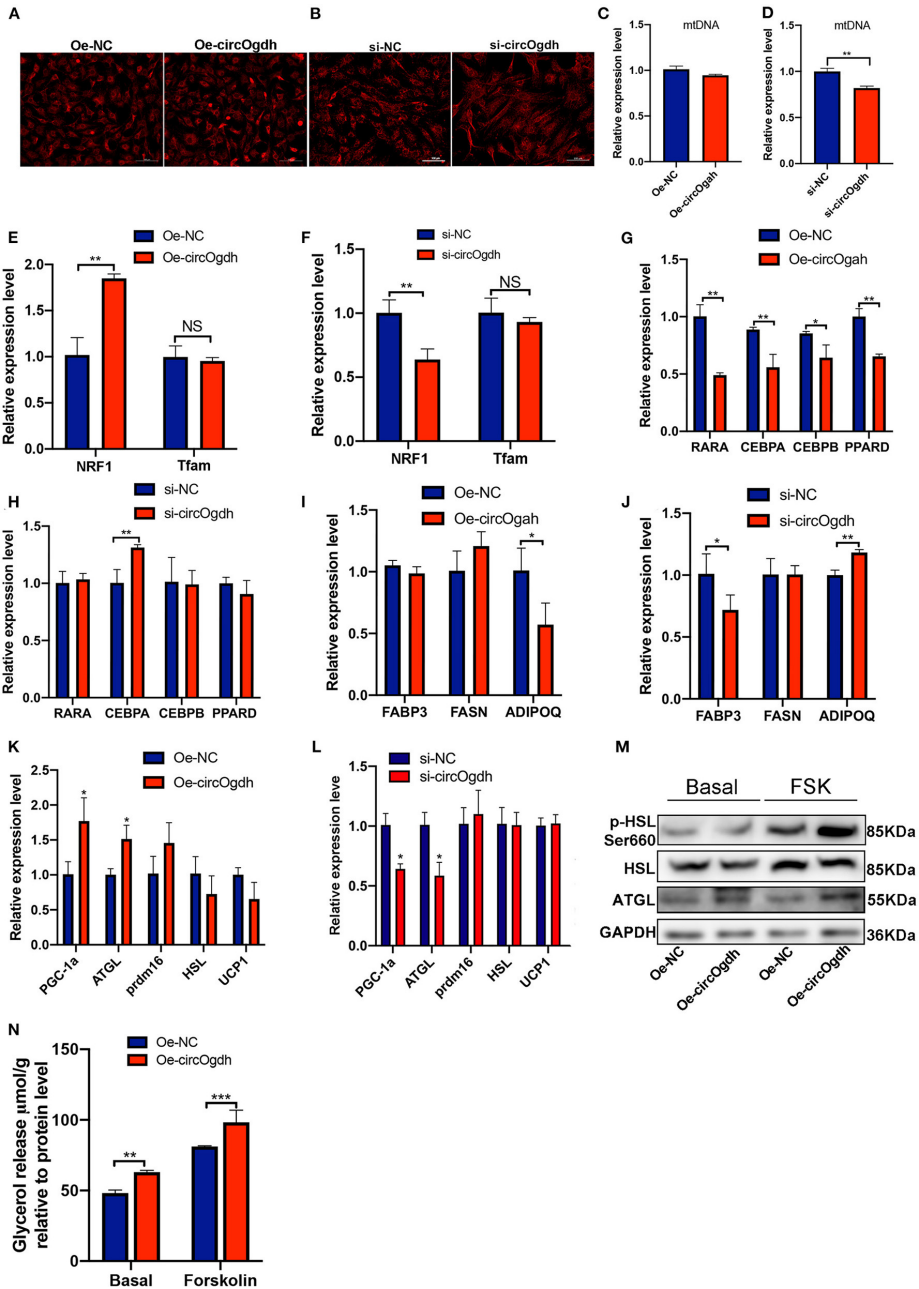
CircOgdh reduces lipid deposition in brown fat by promoting lipolysis after overexpression or knockdown of circOgdh for 48 h, **(A,B)** Mitochondrial membrane potential was assessed by Mito-Tracker Red staining. Scale bar, 100 μm. **(C,D)** Mitochondrial DNA (mtDNA) was detected by one-step fluorescent qPCR. **(E,F)** RT-qPCR detection of mitochondrial-related genes including *NRF1* and *Tfam*. qPCR was used to detect the expression of adipogenesis-related genes **(G,H)**, lipid synthesis-related genes **(I,J)** and lipolysis-related genes **(K,L)**. **(M)** The expression of lipolysis-related proteins, ATGL, HSL, and P-HSL (s660) was detected by western blotting under basal and Fsk stimulation. **(N)** Glycerol content was normalized to the total amount of protein in each sample in the Oe-NC and Oe-circOgdh groups under the basal and Fsk stimulation. All values presented are the mean ± SEM (*n* = 3), *P < 0.05, **P < 0.01, ***P < 0.001, NS, Not Statistically Significant.

To explore the main reason why circOgdh reduces lipid deposition, we first detected the effect of circOgdh on the mitochondrial content of brown fat. Through the detection of Mito-Tracker (Figures 8A,B) and mitochondrial copy number (Figures 8C,D), we found no significant changes in mitochondrial abundance and no significant increase in mitochondrial copy number after circOgdh overexpression. In addition, the detection of mitochondrial biosynthesis-related genes showed that circOgdh could positively regulate the expression of nuclear factor-erythroid 2 related factor 1 (*NRF1*), but not mitochondrial transcription factor A (*Tfam*) (Figures 8E,F). The results suggest that the regulation of mitochondria by circOgdh is uncertain. Subsequently, the expression of adipogenic and lipid synthesis-related genes in brown adipocytes were detected, and the results showed that when circOgdh was overexpressed, the expression of adipogenic-related genes *RXRA, CEBPA, CEBPB*, and *PPARD* and the lipid droplet synthesis-related gene *ADIPOQ* were significantly decreased (Figures 8G,I), However, after circOgdh knockdown, only *CEBPA* and *PPARD* were up-regulated, and *FABP3* was down-regulated (Figures 8H,J). Finally, we examined the expression of selected lipolytic and thermogenic genes, including *Pgc-1a, Atgl, Prdm16, Hsl*, and *Ucp1*. In brown adipocytes overexpressing or knocking down circOgdh, circOgdh positively regulated the expression of *Pgc-1a* and *Atgl* (Figures 8K,L). The detection of glycerol concentration and lipolysis-related proteins, ATGL, HSL, and p-HSL (Ser660) in brown adipocytes showed that the level of lipolysis were significantly increased in the circOgdh overexpression group under basal and forskolin stimulation (Figures 8M,N). The above data suggest that circOgdh can reduce fat deposition by promoting lipolysis.

### CircOgdh acts as an miR-34a-5p sponge to promote brown adipocyte lipolysis

**Figure 9:**
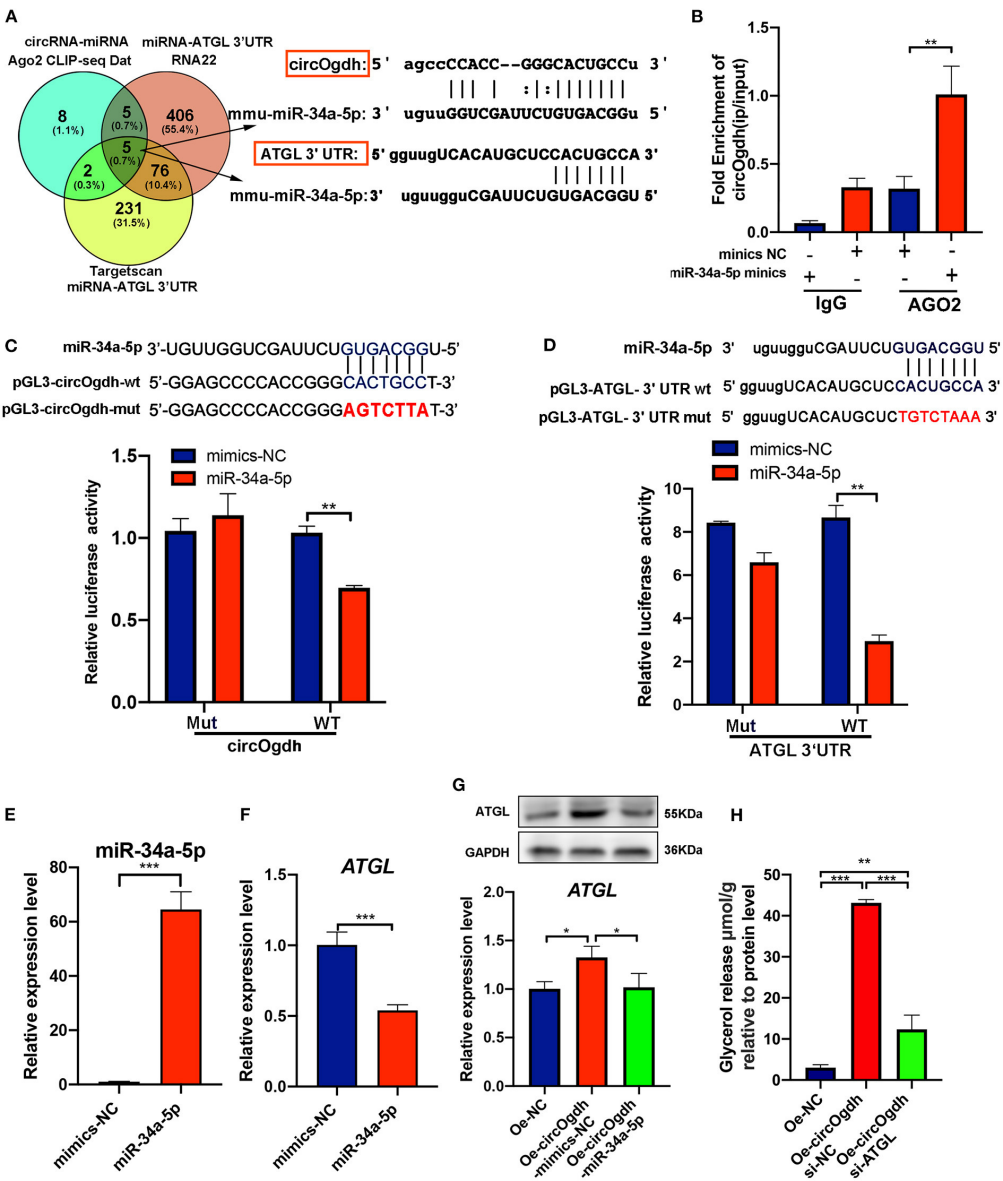
CircOgdh promotes lipolysis by alleviating the miR-34a-5P-mediated inhibition of ATGL expression. **(A)** Ago CLIP-seq data (https://starbase.sysu.edu.cn), RNA22 (https://cm.jefferson.edu/rna22), and Targetscan (https://www.targetscan.org) were used to predict miRNAs targeting both circOgdh and *Atgl*. **(B)** RIP was performed using the AGO2 antibody in BAT cells transfected with miR-34a-5p mimics or NC mimics. CircOgdh enrichment was then detected. **(C,D)** Luciferase reporter activity of circOgdh and *Atgl* 3′UTR in HEK-293T cells co-transfected with miR-34a-5p mimics or NC mimics. **(E,F)** RT-qPCR analysis of miR-34a-5p and *Atgl* in the control and miR-34a-5p-overexpressing brown adipocytes. **(G)** Detection of *Atgl* mRNA and protein expression levels *via* qRT-PCR and western blot after transfection with the control, Oe-circOgdh mimic-NC, or Oe-circOgdh mimic-miR-34a-5p overexpression vector. **(H)** The release of glycerol content was normalized to the total amount of protein for each sample in NC, Oe-circOgdh overexpression and Oe-circOgdh si-ATGL groups under Fsk stimulation. Values in **(B–H)** are means ± SEM (*n* ≥ 3). *P < 0.05, **P < 0.01, ***P < 0.001.

To further explore the mechanism by which circOgdh promotes lipolysis. The miRNAs that interact with circOgdh were predicted based on publicly available Ago CLIP-seq data (https://starbase.sysu.edu.cn), while those that could target *Atgl* were predicted using RNA22 (https://cm.jefferson.edu/rna22) and Targetscan (https://www.targetscan.org). Results were combined to generate a Venn diagram. A total of five miRNAs were found, including mmu-miR-34c-5p, mmu-miR-449a-5p, mmu-miR-34a-5p, mmu-miR-449b, and mmu-miR-760-3p (Figure 9A). Review of the literature indicated that miR-34a-5p is involved in the regulation of brown fat lipid metabolism (23). We conducted AGO2 immunoprecipitation to determine whether circOgdh serves as a scaffold for AGO2 and miR-34a-5p, with the results indicating that circOgdh was specifically enriched in miR-34a-5p transfected cells (Figure 9B).

To confirm that circOgdh and *Atgl* could be regulated by miR-34a-5p, we constructed luciferase reporters containing wild-type and mutated putative binding sites of the circOgdh and *Atgl* transcripts, respectively. Luciferase reporter assays showed that the luciferase activities of the circOgdh and *Atgl* wild-type reporter were significantly reduced under transfection with miR-34a-5p mimics compared to with the control or mutated luciferase reporter (Figures 9C,D). When miR-34a-5p was overexpressed, the mRNA expression of *Atgl* was significantly decreased, indicating that miR-34a-5p could negatively regulate *Atgl* (Figures 9E,F). In addition, we performed co-transfection of circOgdh overexpression vector and miR-34a-5p mimics in brown adipocytes. Overexpression of circOgdh significantly upregulated the mRNA and protein expression of ATGL compared with the control group, while overexpression of the miR-34a-5p mimic significantly suppressed *Atgl* upregulation by circOgdh (Figure 9G). The results suggest that circOgdh can act as an miR-34a-5p sponge to promote *Atgl* expression. Such regulation could be the main reason why circOgdh promotes lipolysis. We further overexpressed circOgdh in brown adipocytes, then knocked down *Atgl* and induced its adipogenic differentiation. Through the detection of glycerol concentration in the medium, it was found that *Atgl* knockdown could counteract the promoting effect of circOgdh on lipolysis and significantly reduce the content of glycerol released into the medium compared with the circOgdh overexpression group (Figure 9H).

## Discussion

WAT and BAT have distinct functions in mammals, with the former being responsible for energy storage, while the latter is mainly implicated in fat decomposition and heat production (24). In the current study, we obtained WAT and BAT from the abdominal subcutaneous and scapular regions of mice, respectively. Studies have shown relatively low numbers of mitochondria in WAT as oppose to a great abundance in BAT, and UCP1 expression was significantly higher in the former, which is consistent with our results (25, 26). These observations indicated that we obtained appropriate adipose tissue samples, which would guarantee the quality of subsequent transcriptome sequencing.

Previous studies demonstrated that various non-coding RNAs, including miRNAs and long non-coding RNAs (lncRNAs), play critical regulatory roles in WAT and BAT (27, 28). The differential lncRNA expression profiles in WAT and BAT were identified *via* lncRNA microarray technology (29). However, the function of circRNA in WAT and BAT adipogenesis has remained elusive. In the present study, we obtained 12,183 circRNAs from WAT and BAT samples, of which 7,477 were identified as novel circRNAs, thus expanding the mouse circRNA database. Consistent with a previous study (30), most circRNAs were derived from protein-coding exons, indicating that circRNAs are generated by the splicing and circularization of precursor mRNAs. The exons of circRNA originating from a single exon were longer than those produced generated from multiple exons. This phenomenon has also been reported in other studies, and it may be that a certain exon length is required for circularization to occur (31). We identified 165 upregulated and 79 downregulated circRNAs between BAT and WAT. Further, 10 DE circRNAs were verified through RT-qPCR, and their relative abundance was consistent with RNA-seq data, suggesting that our sequencing results were reliable. Thus, DE circRNAs could be subjected to further analysis.

CircRNAs regulate host gene expression by competing with linear RNA splicing (22). To explore the role of DE circRNAs in BAT and WAT, we performed GO and KEGG enrichment analyses of DE circRNA host genes. In particular, a number of GO terms related to mitochondria were significantly enriched, including the mitochondrial inner membrane and the mitochondrial envelope. As brown adipocytes specifically dissipate energy in the form of heat through UCP1 (32, 33), they require more mitochondria than white adipocytes. Moreover, DE circRNAs are enriched in signaling pathways related to the browning of white fat and brown fat thermogenesis, such as the AMPK and HIF-1 signaling pathways. Previous findings indicated that AMPK is important for the browning of WAT as well as for controlling mitochondrial quality in BAT (34, 35). Moreover, HIF-1α upregulation in adipose tissue can induce obesity by suppressing BAT thermogenesis (36). Taken together, these data suggest that DE circRNAs regulate WAT browning and the thermogenic capacity of WAT through their host genes.

In the last few years, various studies have described the involvement of non-coding RNAs in the regulation of brown fat differentiation and thermogenesis, such as miR-378, miR-203, and mir-199a-3p (6, 37, 38). The Kemper group reported that downregulation of miR-34a in obese mice increased beige fat markers in WAT and BAT browning. Further, miR-34a could reduce the transcriptional activity of PGC-1a and inhibit browning through the regulation of FGFR1 and SIRT1 (23). Another study showed that miR-124a could promote lipid droplet accumulation by inhibiting the expression of ATGL, while miR-124a inhibition alleviated hepatic lipid deposition by upregulating ATGL/SIRT1 (39). LncRNAs have also been reported as involved in fat browning, with lncRNA TUG1 promoting the browning of white fat through the miR-204/*SIRT1* axis (40). In addition, lncRNA FOXC2-AS1 and LncRNA ROR have also been shown to be involved in white fat browning (41, 42). However, studies on the circRNA-mediated regulation of brown fat differentiation and thermogenesis remain limited.

CircRNAs are a novel class of non-coding RNAs, which are involved in various physiological and pathological processes (43). They exhibit a clear regulatory function in the proliferation and migration of various tumor cells. For example, circ-ERBIN, circ-RanGAP1, circ_001422, and other circRNAs can regulate tumorigenesis through circRNA-miRNA networks (44–46). CircRNAs are also involved in stem cell differentiation, with potential use as novel markers of the process. The circDAB1/miR-1270/miR-944*/*RBPJ/DAB1 axis has been implicated in bone marrow-derived stem cell proliferation and osteogenesis (47). circFOXP1 upregulated *FOXP1 via* miR-33a-5p to promote the osteogenic differentiation of adipose-derived mesenchymal stem cells (48). We found that the DE circRNA circOgdh acts as a sponge for miR-34a-5p, thus regulating brown preadipocyte proliferation and differentiation, adding to knowledge on the mechanisms of circRNA-miRNA network regulation in brown adipocytes.

According to the results of the present study, high expression of circOgdh reduced lipid deposition; however, the phenomenon may not be strongly related with mitochondrial function. In addition, according to our data, high expression of circOgdh does not cause significant changes in mitochondrial membrane potential and mtDNA copy number. The expression level of genes related to lipid synthesis and metabolism, such as NRF1, CEBPA, CEBPB, PPARD, ADIPOQ, and RARA were detected and down-regulated in the circOgdh overexpression group, and the factors may also be responsible for the reduced lipid accumulation. However, when we knocked down circOgdh, circOgdh upregulated the expression of NRF1, CEBPA, and ADIPOQ, but not other genes. Therefore, whether circOgdh reduces lipid content by affecting lipid synthesis remains to be further elucidated. High expression of circOgdh can reduce lipid content in brown adipocytes, at least in part due to the regulation of ATGL by circOgdh, a key enzyme in lipolysis (49, 50). Our results suggest that ATGL tends to promote lipolysis in brown adipocytes, similar to the finding of Albin Hermetter's group (51). Our study also had some limitations. For example, it remains unclear whether circOgdh regulates brown fat development by binding to proteins or other miRNAs, or whether circOgdh regulates browning, which should be investigated further in future research work.

## Conclusions

In this study, we identified a number of DE circRNAs between WAT and BAT, which contribute to the understanding of circRNA-mediated adipose tissue regulation. Furthermore, we demonstrated that circOgdh, which was significantly high expressed in brown fat, regulates brown adipocyte proliferation and lipolysis *via* the miR-34a-5p/ATGL axis. Our data may elucidate novel factors implicated in the circRNA regulation of brown fat-related disease as well as potential therapeutic targets.

## Data availability statement

The datasets presented in this study can be found in online repositories. The name of the repository and accession number(s) can be found at: National Center for Biotechnology Information (NCBI) BioProject, https://www.ncbi.nlm.nih.gov/bioproject/, PRJNA746058.

## Ethics statement

The animal study was reviewed and approved by Ethics Committee of Peking University Shenzhen Hospital.

## Author contributions

AT conceived and designed the experiments. KL and YD performed the experiments. XL analyzed the data. KL and ZL edited and wrote the manuscript. All authors contributed to the article and approved the submitted version.

## Funding

This work was supported by Grants from the National Natural Science Foundation (81901265), Shenzhen Excellent Technological and Innovative Talent Training Project (RCBS20200714114818372), China Postdoctoral Science Foundation (2020M670050ZX), and Shenzhen Science and Technology Plan Project Key Technical Tackling Project (JSGG20200225151806035).

## Conflict of interest

The authors declare that the research was conducted in the absence of any commercial or financial relationships that could be construed as a potential conflict of interest.

## Publisher's note

All claims expressed in this article are solely those of the authors and do not necessarily represent those of their affiliated organizations, or those of the publisher, the editors and the reviewers. Any product that may be evaluated in this article, or claim that may be made by its manufacturer, is not guaranteed or endorsed by the publisher.

## Supplementary material

Supplementary Table S1 Primers used in this study.

Supplementary Table S2 Summary of sequencing data.

Supplementary Table S3 Summary of sequencing data alignment.

Supplementary Table S4 Information of identified circRNAs.

Supplementary Table S5 Differentially expressed (DE) circRNAs.

Supplementary Table S6 GO enrichment analysis of host genes of differentially expressed (DE) circRNAs.
