# Supplementary material for: CircRNA-mediated regulation of brown adipose tissue adipogenesis
Source: Front Nutr. 2022 Jul 29;9:926024. doi: 10.3389/fnut.2022.926024 (PMC9372764; doi:10.3389/fnut.2022.926024)
Supplement: Supplementary Table S1 — Primers used in this study. [file Table_1.DOCX]

**Supplementary Table S1 Primers used in this study**

| CircRNA name | Primer sequences |
| --- | --- |
| mmu_circ_0000865 | F: TGACGTGGAAGCAGTAGACA  R: CAAAATCCTTGGCCAGCTCA |
| mmu_circ_0000231 | F: TACGAGGGCACCATGTAGCA  R: AGGTTGTGCTTCCACCAGG |
| mmu_circ_0005882 | F: AAGTGCTCTGGGTCTGTGCC  R: TCGCTGCTTATGATCTTACTGGTG |
| mmu_circ_0009498 | F: TGAAGGAGCCCAACAGCAAA  R: GCTGAGCGGTGGTTAGGGAG |
| mmu_circ_0006811 | F: TACCAGCCACAGCAGGACAT  R: GGGTGAGTACCCATCCCATACA |
| mmu_circ_0015092 | F: CTGCCACAGAGGAATCCCAG  R: AGGAACAGCGTGACAAGTCA |
| mmu_circ_0000433 | F: AAACTTGCAGATTTTGGACT  R: TAGGAGAAATGCTTGAATGA |
| mmu_circ_0005526 | F: ATGATTCTCCGAATGGTGTC  R: AGAAGGCACAAATCCTGACAA |
| novel_circ_0004306 | F: AGAAGGCACAAATCCTGACAA  R: CAAATGCGTAAATCCCAAGC |
| novel_circ_0000019 | F: GAGAAACTACCACCAAGAGT  R: CTGAGTAACACCAAGGAATC |
| ATGL | F: GGATGGCGGCATTTCAGACA  R: CAAAGGGTTGGGTTGGTTCAG |
| PGC-la | F: TATGGAGTGACATAGAGTGTGCT  R: CCACTTCAATCCACCCAGAAAG |
| UCP1 | F: AGGCTTCCAGTACCATTAGGT  R: CTGAGTGAGGCAAAGCTGATT |
| Prdm16 | F: CCAAGGCAAGGGCGAAGAA  R: AGTCTGGTGGGATTGGAATGT |
| HSL | F: CCAGCCTGAGGGCTTACTG  R: CTCCATTGACTGTGACATC |
| Tfam | ATTCCGAAGTGTTTTTCCAGCA  TCTGAAAGTTTTGCATCTGGGT |
| NRF1 | AGCACGGAGTGACCCAAAC  TGTACGTGGCTACATGGACCT |
| PPARD | TCCATCGTCAACAAAGACGGG  ACTTGGGCTCAATGATGTCAC |
| CEBPB | TGCAGAAGAAGGTGGAGCAG  GCTTGAACAAGTTCCGCAGG |
| RXRA | ATGGACACCAAACATTTCCTGC  CCAGTGGAGAGCCGATTCC |
| FABP3 | ACCTGGAAGCTAGTGGACAG  TGATGGTAGTAGGCTTGGTCAT |
| FABP6 | CTTCCAGGAGACGTGATTGAAA  CCTCCGAAGTCTGGTGATAGTTG |
| ADIPOQ | TGTTCCTCTTAATCCTGCCCA  CCAACCTGCACAAGTTCCCTT |
| GAPDH | F: ATCACTGCCACCCAGAAGACT  R: CATGCCAGTGAGCTTCCCGTT |
| mmu-miR-34a-5p | F: TGGCAGTGTCTTAGCTGGTTGT |
| siRNA-circOgdh | Sense: AGACAAACTTGTCATGGGA |
| siRNA-ATGL | Sense: AAGTTCATTGAGGTATCTA |
